# Retroperitoneal Immature Teratoma in a Neonate

**DOI:** 10.21699/jns.v6i2.586

**Published:** 2017-04-15

**Authors:** Chiranjiv Kumar, Rajpal Singh Sisodiya, Shasanka Shekhar Panda, Yogesh Kumar Sarin

**Affiliations:** Department of Paediatric surgery, Maulana Azad Medical College and Lok Nayak hospital, New-Delhi-110002

**Keywords:** Retroperitoneum, Immature, Teratoma

## Abstract

We report a rare case of large immature retroperitoneal teratoma in a neonate. The diagnostic and therapeutic challenges of dealing with such a case have been discussed and the relevant literature reviewed.

## CASE REPORT

A 14-day-old female neonate, born at term, product of non-consanguineous marriage with normal antenatal scan and an uneventful pregnancy, was admitted with complaint of progressively increasing mass in left abdomen for 5 days. Abdominal examination revealed single nonmobile, non-peristaltic, non-pulsatile, non-compressible mass with variegated consistency and involving left lumbar, left hypochondrium, left iliac fossa and umbilical region, crossing the midline 2cm on the right side. 

**Figure 1:**
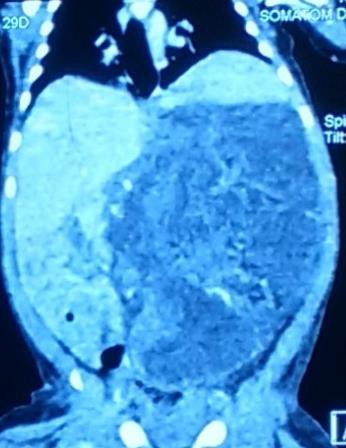
CECT abdomen revealed a large relatively well-defined heterogeneously enhancing mass lesion involving the entire left half of abdomen with calcification.

Haematological investigations were normal. Serum alpha fetoprotein was 3338 IU/ml, and β-human chorionic gonadotrophins as 0.136 IU/ml. Her 24 hours urinary vanillyl mandellic acid was also normal. Plain x-ray showed bowel gas shadow pushed to right side by large space occupying lesion on left side of abdomen with calcification. Ultrasonography of abdomen showed large solid and cystic mass in left abdomen with internal vascularity, the left kidney was not seen separately. Contrast enhanced computed tomography of abdomen revealed a large relatively well-defined heterogeneously enhancing mass lesion of size 13x 9.5x 9 cm, involving the entire left half of abdomen (Fig. 1). The lesion showed scattered areas of calcific and fat attenuation and displacing surrounding viscera. However, no encasement was seen. Inferiorly, the lesion was reaching till the pelvic inlet and causing inferior displacement and malrotation of left kidney which was otherwise normal. USG-guided FNAC revealed few small clusters of polygonal cells with moderately abundant pale blue/ greyish cytoplasm and central nuclei. Smear also showed fair number of scattered histiocytes, fibroblasts and fat cells with occasional cluster of mesothelial cells. A possibility of mature cystic teratoma was suggested.

**Figure 2:**
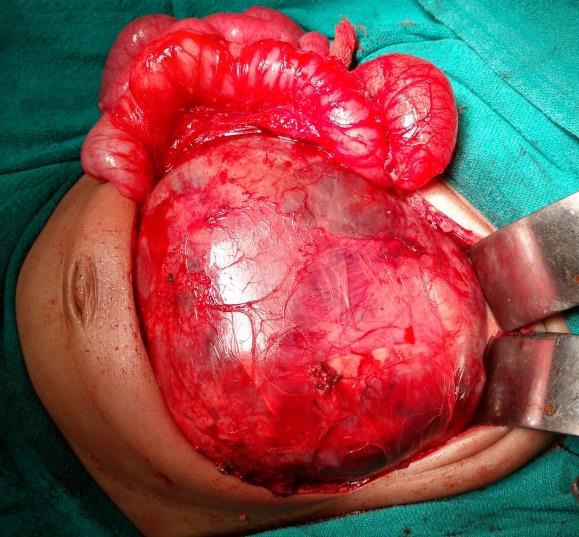
Retroperitoneal teratoma engulfing left kidney.

Intraoperatively, a large solid cystic mass was engulfing the left kidney (Fig. 2). Total excision of mass along with left nephroureterectomy, and lymph node sampling was done. Ascitic fluid cytology showed only reactive mesothelial cells in a blood mixed hemorrhagic background. Cut section of the excised specimen revealed a variegated grey white to gelatinous and hemorrhagic tumour replacing nearly entire kidney with preserved focal rim of normal parenchyma. Histopathology revealed it to be immature teratoma grade-II. The tumour had invaded the renal pelvis, though the pelvic mucosa was not breached. Adrenal gland was free of tumour. Excised hilar lymph nodes were free of tumor. Immature component was neuroepithelium admixed with mainly mature component along with few immature cartilage and skin; no yolk sac component was identified. 

The post-operative period was uneventful. The patient has been doing well on 7 months follow-up with no clinical, radiological and biochemical evidence of any recurrence. 

## DISCUSSION

Incidence of retroperitoneal teratoma is bimodal with peaks in the first 6 months of life and in early adulthood [1, 2]. Retroperitoneal teratoma constitute 3.5-4% of all germ cell tumors in children and 1-11% of primary retroperitoneal tumours [3,4]. Three cases of neonatal retroperitoneal teratoma was reported by Lack *et al*. [5]; of these, two were immature and both succumbed to the disease. The presence of nephroblastic components is extremely rare in retroperitoneal teratomas [6]. It is also difficult to differentiate retroperitoneal immature teratoma from retroperitoneal neurogenic and yolk sac tumors, which occurs mostly in the axis of the body [7].

The prognosis of neonatal teratoma is favorable with an 80-100% survival reported after surgical excision of the tumor and treatment of any recurrence [4]. Adjuvant chemotherapy was not given in the index case as being a neonate and there was no tumour spillage intraoperatively. However, for incomplete surgical excision and for recurrence patient should receive 4-6 cycles of platinum based chemotherapy [2]. To conclude, excision of large retroperitoneal teratoma in a neonate can be a difficult surgical endeavour, however long-term outcome must be evaluated very closely.

## Footnotes

**Source of Support:** None

**Conflict of Interest:** None
